# Calcipotriol-betamethasone foam modulates serum and tissue IL-17A, IL-31, IL-31RA, and substance P in mild-to-moderate psoriatic itch: A clinical and immunopathological study

**DOI:** 10.1016/j.xjidi.2026.100541

**Published:** 2026-09-21

**Authors:** Irene Bonanni, Lavinia Quintarelli, Elena Del Bianco, Beatrice Bianchi, Marta Donati, Alessandro Magnatta, Luca Sanna, Virginia Corti, Simone Landini, Elena Biancamaria Mariotti, Valentina Ruffo di Calabria, Alberto Corrà, Alessio Coi, Chiara Della Bella, Alice Verdelli, Walter Volpi, Marzia Caproni

**Affiliations:** 1 Rare Skin Diseases Unit, Section of Dermatology, Department of Health Sciences, European Reference Network for Rare and Undiagnosed Skin Disorders Member, University of Florence, Florence, Italy; 2 Section of Dermatology, Department of Health Sciences, University of Florence, Italy; 3 Department of Dermatology, University of Modena and Reggio Emilia, Modena, and Azienda Unità Sanitaria Locale - Istituto di Ricovero e Cura a Carattere Scientifico di Reggio Emilia, Skin Cancer Centre, Reggio Emilia, Italy; 4 Dermatology Unit, Azienda Unità Sanitaria Locale Toscana Nord Ovest, Lucca, Italy; 5 Dermatology Unit, Ospedale San Bartolo, Vicenza, Italy; 6 Institute of Clinical Physiology, National Research Council, Pisa, Italy; 7 Department of Molecular and Developmental Medicine, University of Siena, Siena, Italy

**Keywords:** IL-31, Itch, PASI, Psoriasis, p-VAS

## Abstract

Although itching is a common psoriasis symptom, its pathogenesis remains partially elucidated. We evaluated clinical pruritus features and associated serum and tissue cytokine profiles in 25 patients with mild-to-moderate psoriasis who were treated with calcipotriol-betamethasone foam over 4 weeks. Clinical scores comprised the PASI, Dermatology Life Quality Index, pruritus Visual Analogue Scale, and Itch Severity Scale. Serum and tissue levels of IL-17A, IL-31, IL-31RA, and substance P were evaluated before and after therapy. Most patients had severe pruritus and marked QOL impairment. PASI was significantly related to Dermatology Life Quality Index, unlike itch scores. Significant sex-related differences in itching scores emerged, while Body Mass Index and lesion localization were associated with specific clinical scores. Treatment induced a marked reduction of cytokines in both serum and lesional tissue, although no correlation was found with clinical scores. Immunohistochemistry and immunofluorescence demonstrated distinct cutaneous distributions for each mediator, suggesting an interplay of neural and immune pathways, with eccrine glands as a potential anatomical hub. Our findings suggest a local modulation of the cutaneous neuro-immune axis by calcipotriol-betamethasone foam. Ultimately, given the topical nature of this therapy, tissue-level measurements provide a more accurate representation of lesional pruritus dynamics than serological data.

## Introduction

Psoriasis is a chronic immune-mediated dermatosis that primarily affects the skin, with an estimated prevalence of 3% in the general population. Historically considered non-pruritic, recent studies have reported itch in 65–98% of patients (Pithadia et al, 2020; Prignano et al, 2009; Szepietowski et al, 2004; Yosipovitch et al, 2000). Despite its prevalence and significant impact on QOL, the pathogenesis of psoriatic itch remains unclear, likely involving a complex interplay of immune, neural, endocrine, and vascular mechanisms (Jaworecka et al, 2021; Pithadia et al, 2020).

The immune system drives pruritus via multiple mediators, amplifying local inflammation. IL-31 signals through the IL-31RA/oncostatin M receptor complex, which is constitutively expressed by stromal and epithelial cells, keratinocytes, and a specific subset of itch-sensitive neurons (Bilsborough et al, 2006; Cevikbas et al, 2014; Dillon et al, 2004). In keratinocytes, IL-31RA activation promotes proinflammatory signaling, itch pathways, epidermal thickening, barrier dysfunction, and angiogenesis (Cornelissen et al, 2011; Feld et al, 2016; Furue et al, 2018). Elevated IL-31 serum levels and lesional IL-31/IL-31RA expression have been reported in psoriasis and decrease following therapies including narrowband UVB, anti-TNFα, nemolizumab, and tofacitinib (Kabashima and Irie, 2021; Karamova et al, 2024; Narbutt et al, 2013; Purzycka-Bohdan et al, 2021; Sonkoly et al, 2006).

Key cytokines driving psoriasis pathogenesis, like IL-17A, also mediate itch via dorsal root ganglion neurons, and anti-IL-17A mAbs have shown antipruritic effects in patients with psoriasis (Bautista-Herrera et al, 2020; Kimball et al, 2018; Moynes et al, 2014).

Tachykinins, such as substance P (SP), play a pivotal role in pruritus. Intradermal SP injection induces itch in healthy skin, and psoriatic lesions exhibit elevated SP, receptor expression, and SP-immunoreactive fibers (Amatya et al, 2011; Naukkarinen et al, 1989; Reich et al, 2007). Furthermore, SP degranulates mast cells, activates lymphocytes, and promotes epithelial and connective tissue proliferation (Chan et al, 1997; Foreman, 1987). Therapies including narrowband UVB, evodiamine, and botulinic toxin reduce SP levels in serum and lesions (Aschenbeck et al, 2018; Liang et al, 2024).

The present study aimed to assess, prospectively, the clinical and immunopathological features of pruritus in patients with mild-to-moderate psoriasis treated with calcipotriol-betamethasone foam. Disease severity was evaluated using the PASI, whereas pruritus was investigated using the Dermatology Life Quality Index (DLQI), Visual Analogue Scale for pruritus (p-VAS), and Itch Severity Scale (ISS). Specifically, we analyzed serum and tissue levels of IL-17A, IL-31, IL-31RA, and SP to assess their variations pre- and posttherapy and investigated the correlations with clinical scores and demographic features such as Body Mass Index (BMI) and sex. Given the scarcity of literature on local mediator topography, we also performed an exploratory analysis of their cutaneous distribution to clarify the localized interplay between neural, immune, and pruritus-specific pathways during topical treatment.

## Results

In a cohort of 12 females and 13 males (sex ratio = 1.08), patients were identified as Asian (n = 1) or White (n = 24). The average disease onset and diagnosis were at a median of 51.0 and 53.4 years, respectively, with a median diagnostic gap of 7 months. The average BMI was 25.97 kg/m^2^, and patients were stratified into 3 BMI categories: normal weight (18.5–24.9 kg/m^2^), overweight (25.0–29.9 kg/m^2^), and obese (≥30.0 kg/m^2^). The associations between BMI, clinical scores, and serum cytokine levels at baseline and after treatment are summarized in Table 1. A family history of psoriasis was found in 48.0% of cases (12/25). Hypertension and dyslipidemia were the most frequent comorbidities, along with their respective therapies. Clinically, the scalp and upper limbs were the most affected (both at 60%, 15/25), followed by the lower limbs (56%, 14/25), torso (32%, 8/25), and other localizations (20%, 5/25). As shown in Table 2, PASI correlated with the scalp and lower limbs (*P* = .012 and .025, respectively), the upper limbs correlated with DLQI (*P* = .046) and p-VAS scores (*P* = .042), while the trunk showed no significant association.

**Table 1:** Association Between BMI, Clinical Scores, and Serum Cytokines at Baseline and After Treatment

BMI	PASI	DLQI	p-VAS	ISS	IL-17A	IL-31	SP
**Normal weight**	3.7 [2.7–6.2]	5 [2.0–8.0]	4.90 ± 3.57	3.79 ± 1.96	21.33 ± 3.59	90.6 [62.8–624]	401 [310–458]
**Overweight**	2.4 [1.0–7.0]	5 [1.0–7.0]	7.29 ± 1.73	5.21 ± 2.67	22.37 ± 3.40	376 [63.8–1030]	408 [396–453]
**Obese**	4.4 [1.8–7.9]	9 [2.0–18.0]	7.00 ± 3.42	3.35 ± 1.94	22.95 ± 3.73	645.7 [28.30–2100]	516.5 [449–738]
***P* value**	.748	.501	.239	.289	.647	.828	.036

**BMI**	**PASI Δ**	**DLQI Δ**	**p-VAS Δ**	**ISS Δ**	**IL-17A Δ**	**IL-31 Δ**	**SP Δ**

**Normal weight**	3.28 ± 1.92	1.89 ± 3.26	3.25 ± 2.82	1.76 ± 1.38	0.06 ± 3.22	37.8 [74–64.3]	111.62 ± 141.58
**Overweight**	3.02 ± 1.88	2.86 ± 3.62	5.21 ± 1.55	4.14 ± 1.90	2.04 ± 2.12	59.56 [3.34–291.80]	93.00 ± 85.39
**Obese**	2.92 ± 2.17	1.83 ± 4.01	4.40 ± 3.65	1.45 ± 0.96	3.20 ± 2.50	161.1 [1.67–260.10]	151.60 ± 119.43
***P* value**	.959	.875	.386	.021	.148	.430	.704

The upper section reports baseline clinical scores and serum cytokine levels, whereas the lower section reports treatment-induced changes (Δ, T1–T0). BMI categories were defined as normal weight (18.5–24.9 kg/m^2^), overweight (25.0–29.9 kg/m^2^), and obese (≥30.0 kg/m^2^). *P* values were calculated using 1-way ANOVA for normally distributed variables and the Kruskal–Wallis test for non-normally distributed variables. Data are presented as mean ± SD or median [25th–75th percentile], as appropriate.

Abbreviations: BMI, body mass index; DLQI, dermatology life quality index; ISS, itch severity score; p-VAS, visual analogue scale for pruritus; SP, substance P.

**Table 2:** Association Between Clinical Scores and Anatomical Site

	Scalp			Lower limbs		
	Involved	Uninvolved	*P* value	Involved	Uninvolved	*P* value
**PASI**	6.00 [2.70–7.90]	2.40 [1.40–3.00]	.013	6.00 [2.70–7.90]	2.10 [1.40–4.00]	.026
**DLQI**	4.50 [2.00–7.00]	8.00 [2.00–13.00]	.303	7.00 [2.00–11.00]	4.00 [2.00–7.00]	.331
**p-VAS**	6.67 ± 3.18	5.4 ± 3.81	.374	5.77 ± 3.74	6.94 ± 1.91	.392
**ISS**	4.54 ± 2.42	3.39 ± 1.79	.253	4.03 ± 1.64	4.24 ± 2.90	.829

	**Upper limbs**			**Trunk**		

	**Involved**	**Uninvolved**	*P* value	Involved	Uninvolved	*P* value

**PASI**	5.20 [2.40–6.30]	2.70 [1.80–6.00]	.271	3.70 [2.70–7.90]	3.20 [1.80–6.00]	.325
**DLQI**	7.00 [4.00–11.00]	2.00 [1.00–7.00]	.042	5.00 [3.00–9.00]	6.00 [2.00–10.00]	.975
**p-VAS**	7.38 ± 2.22	4.82 ± 3.66	.046	5.69 ± 3.35	6.47 ± 3.17	.582
**ISS**	4.50 ± 1.83	3.58 ± 2.76	.361	3.82 ± 1.85	4.26 ± 2.45	.667

Scalp and lower limb involvement were associated with PASI, while upper limbs were associated with DLQI and p-VAS. No significant association was observed between trunk involvement and any clinical score. *P* values were obtained using the *t*-test for p-VAS and ISS and the Wilcoxon signed-rank test for PASI and DLQI. Results are reported as median [25th–75th percentile] for non-normally distributed variables (PASI and DLQI) and mean ± standard deviation for normally distributed variables (p-VAS and ISS).

Abbreviations: DLQI, dermatology life quality index; ISS, itch severity score; p-VAS, visual analogue scale for pruritus.

Median PASI decreased from 4.71 at T0 to 1.20 at T1. Most patients had mild-to-moderate psoriasis (91.7% and 22/24; PASI <10), while 8.3% (2/24) had severe disease (PASI >10) (Mrowietz et al, 2011). DLQI declined from 6.52 at T0 to 1.32 posttreatment. Using Finlay and Khan (1994) thresholds, psoriasis caused significant QOL impairment in 25% (6/24), moderate impairment in 33.3% (8/24), mild impairment in 20.8% (5/24), and minimal impairment in 20.8% of cases (5/24; DLQI <2). Mean p-VAS decreased from 6.15 to 2.05. According to Reich et al (2012) cut-offs, pruritus severity was none-to-mild (20.8%, 5/24), moderate (16.7%, 4/24), and severe (62.5%, 15/24), with one-third reporting very severe itch (p-VAS ≥ 9). ISS declined from 3.07 at T0 to 1.20 at T1. The correlation analysis revealed a moderate significant association between PASI and DLQI (rho = 0.45, *P* = .031), but no correlation with p-VAS or ISS. Females reported higher baseline pruritus than males (p-VAS: 8.04 ± 2.08 vs 4.65 ± 3.17, *P* = .006; ISS: 5.20±2.21 vs 2.83±1.54, *P* = .01), while PASI and DLQI showed no sex-related differences (*P* = .603 and .006, respectively).

In 20 serum samples, IL-17A (*t*-test *P* = .014) and SP (*t*-test, *P* < .0003) levels significantly decreased from T0 to T1, with average differences of −1.67 and −95.24, respectively. IL-31 also declined in most patients (16/20, Wilcoxon *P* = .014).

In tissue samples, IL-17A (*t-*test *P*-value < .0001; average difference of −42.27) and SP (*t-*test *P*-value < .006; average difference of −7.23) decreased significantly, as did IL-31 and IL-31RA (Wilcoxon *P* = .001). A comprehensive summary of data on clinical scores, serum, and immunohistochemical analysis is provided in Table 3. IL17^+^ lymphocytes were diffusely distributed in the superficial to mid-dermis and dermal papillae, with an additional perivascular pattern. Focal epidermal positivity was observed in exocytosed lymphocytes and parakeratotic areas (Figure 1). In lesional skin, IL-31 expression was predominant within the dermal inflammatory infiltrate localized in the superficial dermis, deep perivascular, and papillary areas, along with adnexal regions. In the epidermis, positivity was moderate and scattered among exocytic lymphocytes. Conversely, both perilesional and healthy skin yielded negative results (Figure 2). In the epidermis, IL-31RA expression in keratinocytes gradually decreased from the basal to the corneal layer. In the dermis, slight positivity was observed within the inflammatory infiltrate of the superficial dermis, perivascular, and papillary regions. IL-31RA positivity was also detected in association with eccrine sweat glands and adnexal structures. IL-31RA was absent in perilesional and healthy skin (Figure 3). As for IL-31, SP was intensely spotted within the inflammatory infiltrate, colocalizing with lymphomonocytic cells in the dermal papillae and perivascular dermis, with less frequent distribution in the epidermis, as demonstrated by both immunohistochemistry and immunofluorescence (Figure 4). However, the present analyses do not allow definitive identification of the cellular source of SP, which may include both neural fibers and inflammatory cell populations. In accordance with quantitative analysis, posttreatment images show a markedly reduced staining in all cases. As represented in Figure 5, we further analyzed the localization and the overlap between IL-31, IL-31RA, and CD4^+^ T-cells by fluorescence on confocal microscopy, demonstrating a colocalization between CD4^+^ T lymphocyte positivity and IL-31/31RA expression.

**Table 3:** Clinical and Immunopathological Results at Baseline and After Treatment

Score	T0	N0	T1	N1	Δ	*P*-value Δ	N Δ
PASI	4.71 [1.80–7.90]	24	1.20 [0.10–1.60]	21	−3.50	< .0001	21
DLQI	6.52 [2.00–11.00]	23	1.32 [0.00–3.00]	20	−4.45	.0003	20
p-VAS	6.15 [4.00–8.00]	23	2.05 [0.00–4.00]	19	−4.83	.0002	19
ISS	3.07 [1.51–4.51]	21	1.20 [0.00–2.33]	16	−1.87	.0012	16

**Serum**	**T0**		**T1**		***P*-value Δ**		

IL-17A	21.95 [19.85–24.23]		20.30 [19.55–21.45]		.014		
IL-31	367.45 [63.55–853.90]		335.10 [26.47–616.55]		.014		
SP	432.00 [393.50–462.75]		327.00 [284.5–456.00]		.003		

**Tissue**	**T0**		**T1**		***P*-value Δ**		

IL-17A	73 [49.67–94.17]		30 [11.33–47.67]		.0001		
IL-31	33 [29.17–49.83]		16 [10.33–25.33]		.001		
IL-31RA	27 [11.33–38.34]		9 [1.33–23.33]		.001		
SP	13 [6.00–18.50]		2 [1.00–9.00]		.006		

Schematic results upon PASI, DLQI, p-VAS, and ISS scores at baseline (T0) and after treatment (T1), along with serum and tissue cytokines variations. On serum and tissue, data were available for 20 and 11 patients, respectively. *P*-values were obtained by *t-*test (IL-17A and SP) and Wilcoxon signed-rank tests (IL-31 and IL-31RA). Results are expressed as medians [25th-75th percentile].

Abbreviations: DLQI, dermatology life quality index; ISS, itch severity score; p-VAS, visual analogue scale for pruritus; SP, substance P.

**Figure 1:**
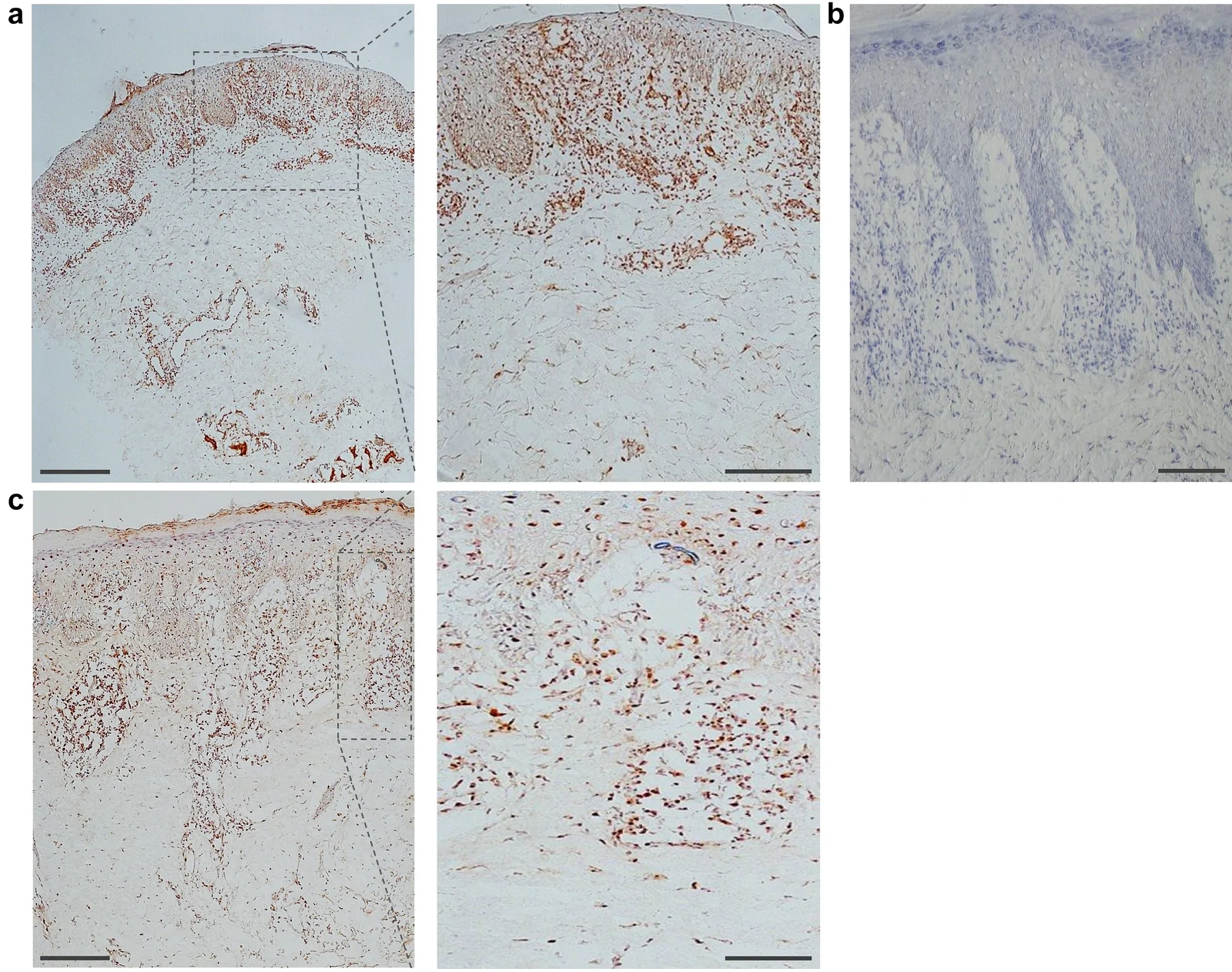
**Immunohistochemical staining of IL-17A.** (**a**) Baseline staining showing predominant immunoreactivity of IL-17A^+^ lymphocytes in the dermal papillae and mid-superficial dermis, with a focal distribution in exocytosed lymphocytes and parakeratotic areas in the epidermis. **(b)** Isotype control. **(c)** Post treatment IL-17A staining demonstrating a marked reduction in immunoreactivity in both keratinocytes and dermal inflammatory infiltrates. Main images were captured at 4× magnification (scale bar 1000 μm), and insets at 10× (scale bar 500 μm). Isotype images were captured at 10× magnification (scale bar 500 μm).

**Figure 2:**
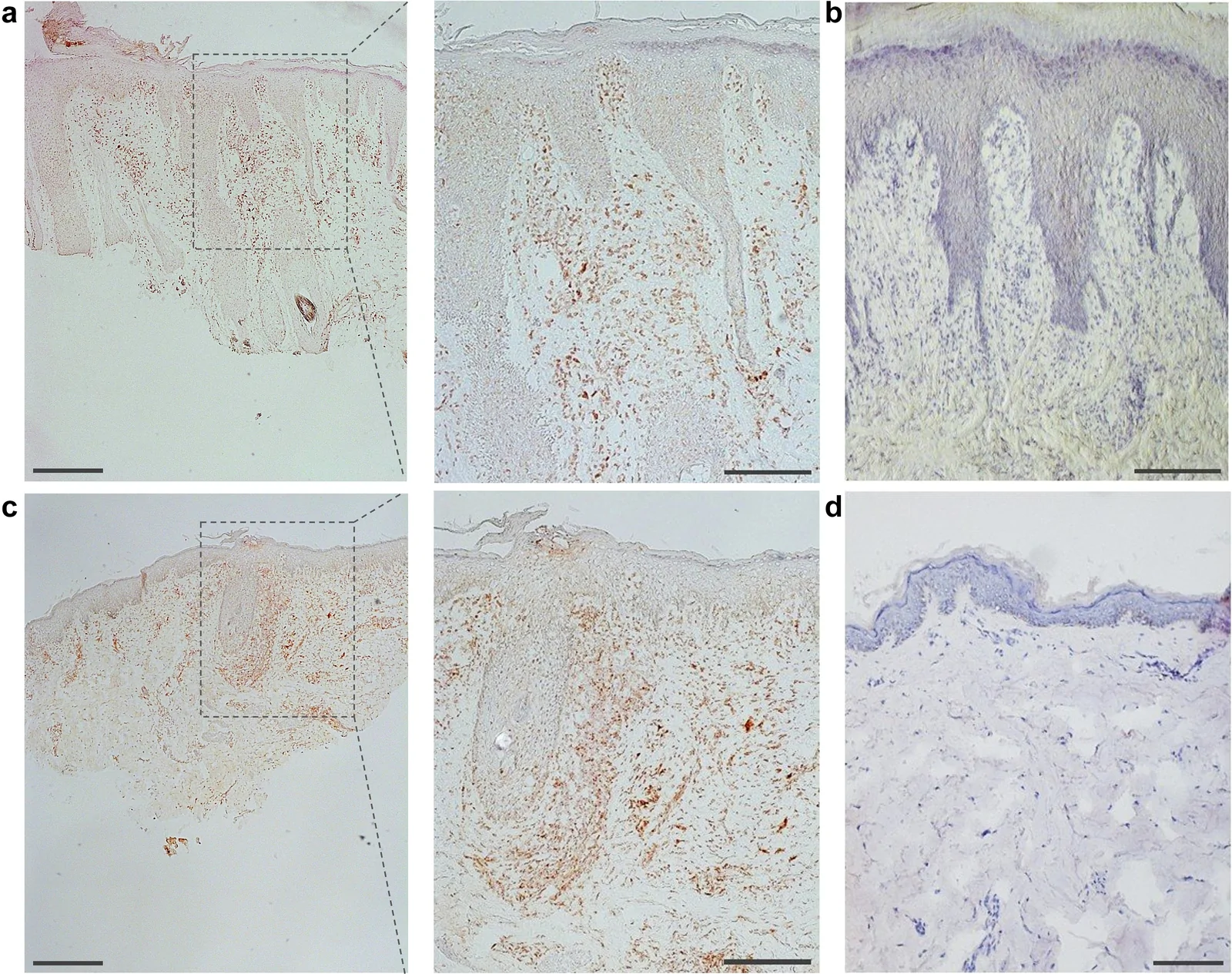
**Immunohistochemical staining of IL-31.** (**a**) At baseline, moderate cytoplasmic staining of keratinocytes is observed in the epidermis of lesional skin, associated with intense staining of the inflammatory infiltrates in both the superficial and deep dermis, as well as appendages. **(b)** Isotype control. **(c)** Immunohistochemical staining of lesional skin at T1 demonstrates a marked reduction in inflammatory infiltrates, accompanied by a decrease in dermal cellularity and immunoreactivity. Epidermal hyperplasia is attenuated, with reduced acanthosis and partial restoration of rete ridge architecture. **(d)** Perilesional skin. In panels **(a)** and **(b)**, representative images were acquired at 4× magnification (scale bar 1000 μm) with insets at 10× (scale bar 500 μm). Isotype images were captured at 10× magnification (scale bar 500 μm), whereas perilesional skin was captured at 4× magnification (scale bar 1000 μm).

**Figure 3:**
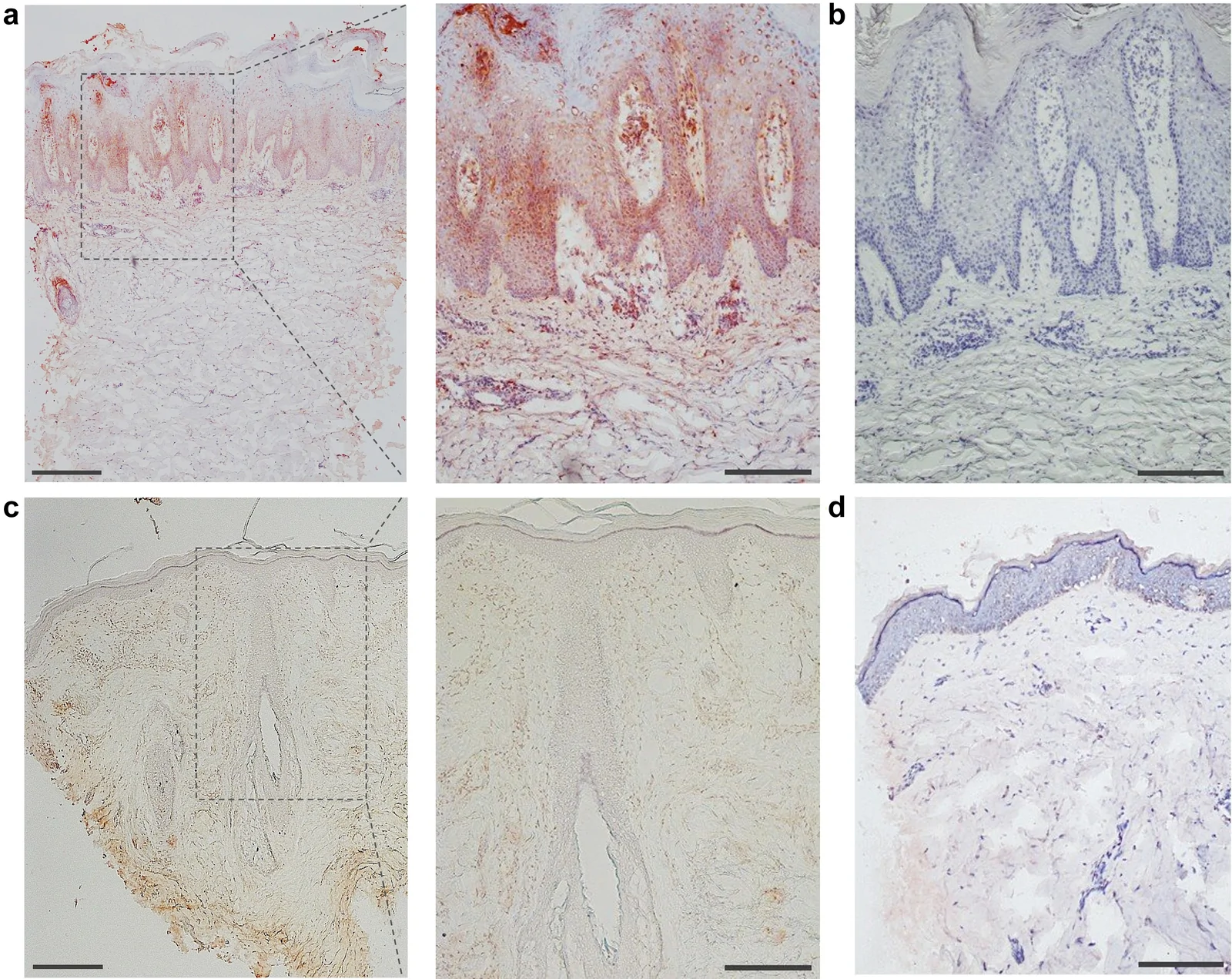
**Immunohistochemical staining of IL-31RA.** (**a**) At baseline, IL-31RA expression in lesional tissue is mainly detected in inflammatory infiltrates of the superficial and deep perivascular dermis, dermal papillae, adnexal structures, and perivascular areas. In the epidermis, staining intensity increases progressively from the basal layer towards the stratum corneum. **(b)** Isotype. **(c)** Significant reduction of IL-31RA immunohistochemical staining in lesional skin after treatment. **(d)** Perilesional skin. Main images were captured at 4× magnification (scale bar 1000 μm), and insets at 10× (scale bar 500 μm). Isotype images were acquired at 10× magnification (scale bar 500 μm), whereas perilesional skin was captured at 4× magnification (scale bar 1000 μm).

**Figure 4:**
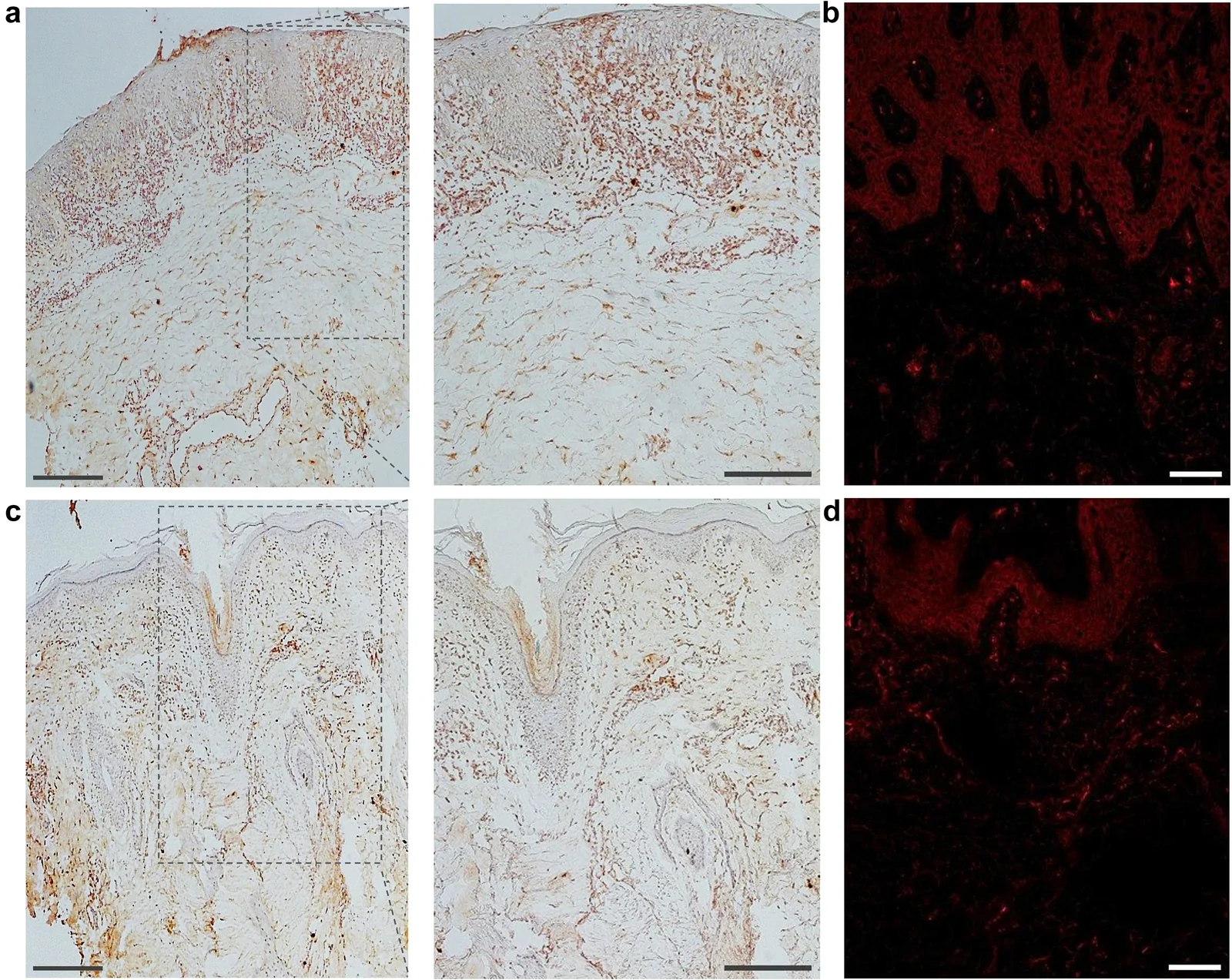
**Substance P staining by immunohistochemistry and immunofluorescence.** (**a**) Baseline immunohistochemistry and **(b)** immunofluorescence of lesional skin, showing SP staining predominantly colocalizing with lymphomonocytic cells in the dermal papillae and perivascular dermis, with limited epidermal involvement. **(c**) Post treatment immunohistochemical and **(d)** immunofluorescence staining, both show a marked reduction in dermal positivity. At immunohistochemistry, representative images were captured at 4× magnification (scale bar 1000 μm), and insets at 10× (scale bar 500 μm). At immunofluorescence, images were acquired both at 20× magnification (scale bar 100 μm).

**Figure 5:**
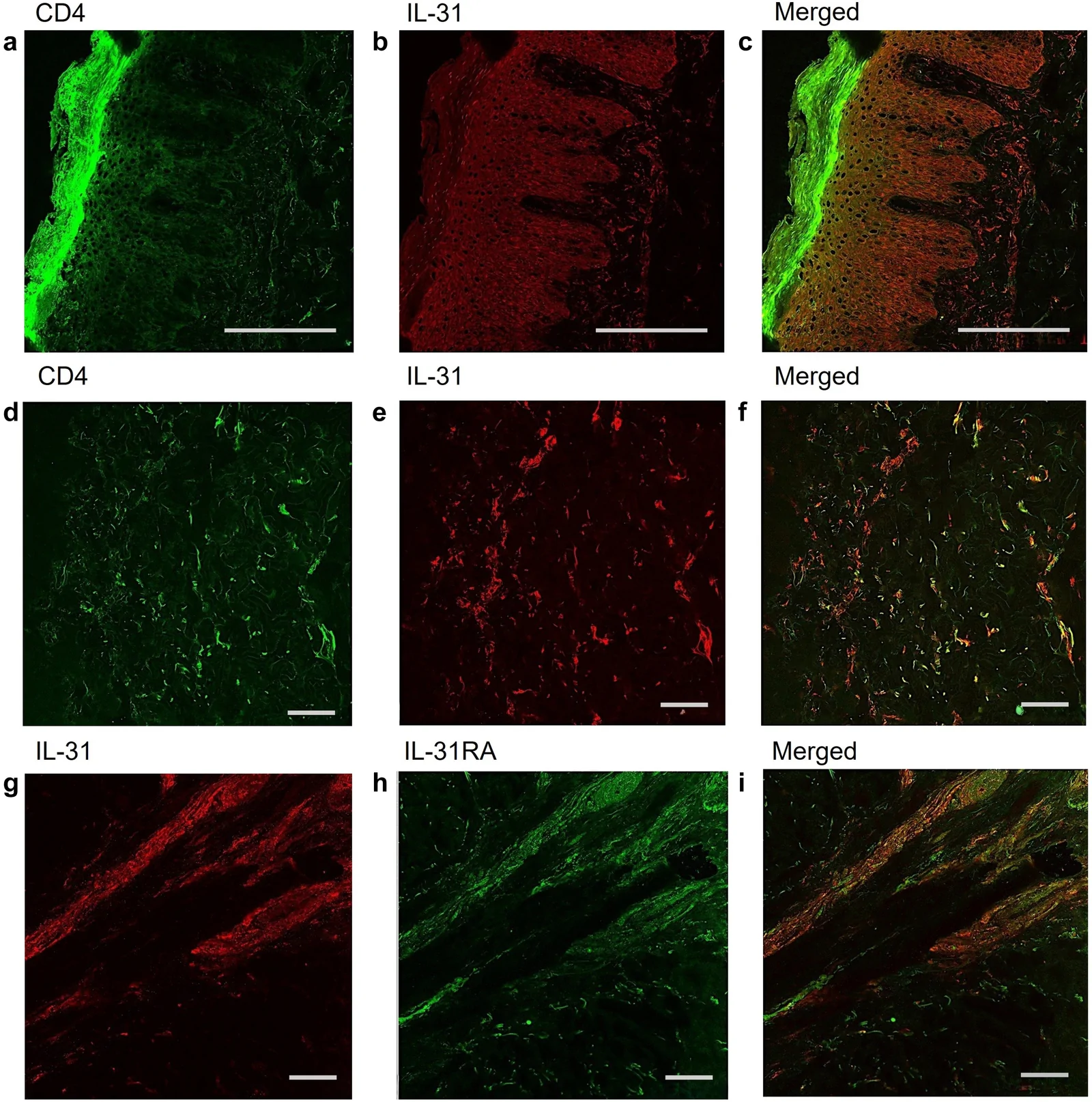
**Confocal microscopy analysis of CD4^+^ T-cells and IL-31/IL-31RA expression.** Epidermic and dermal images were captured at 10× and 40 × magnification, respectively (scale bar 500 and 50 μm, respectively). The first 2 columns show single-channel staining, while the third column represents merged images. **(a–c)** Overlap between CD4^+^ T-cells (green) and IL-31 (red), mostly observed in the epidermis, with lower expression in the dermis. **(d–f)** Dermal colocalization of CD4^+^ T-cells (green) and IL-31 (red). **(g–i)** Expression of IL-31 (red) and IL-31RA (green), mainly localized in the perivascular areas and adnexal structures and detected less in the epidermis and upper dermis.

Ultimately, linear regression did not show a significant statistical association between the reduction in clinical scores and cytokine levels in serum and lesional tissue, even when adjusted for potential confounders such as sex and age at diagnosis.

## Discussion

Although long considered non-itching, psoriasis has a remarkable association with pruritus, significantly impairing patients’ QOL. Multiple mechanisms drive itch pathogenesis, which remains only partly elucidated. Among these, immune mediators play a central role, including both itch-specific (eg, IL-31/IL-31RA) and psoriasis-associated cytokines (IL-17), while neuropeptides, particularly SP, are increasingly recognized as important contributors to initiating and sustaining itch. To deepen our understanding of these mediators, we evaluated the cutaneous distribution and serum levels of IL-17A, IL-31, IL-31RA, and SP in a cohort of patients receiving a 4-week treatment with calcipotriol-betamethasone foam, currently the first-line topical therapy in Italy.

At baseline, females were on average older than males (58.3 vs 42.0 years old), and two-thirds of patients developed late-onset psoriasis (>40 years), typically associated with a milder clinical course and a reduced familial predisposition.

Despite some conflicting data, including our own findings (Czarnecka-Operacz et al, 2015; Mansouri et al, 2019; Nguyen and Tran, 2025), several studies suggest that BMI is associated with pruritus in psoriasis (Bahali et al, 2017; Junsuwan et al, 2023; Mansouri et al, 2019; Morariu et al, 2024), indicating that obesity may promote a proinflammatory state, potentially enhancing itch. In our cohort, BMI was not associated with baseline cytokine levels or clinical scores, except for SP which showed a higher median value in the obese group (*P* = .036). Although both clinical scores and cytokine levels decreased after treatment, only ISS showed a significant association with BMI in terms of treatment-related variation. Overall, these findings may suggest a limited but selective influence of BMI on specific clinical and inflammatory outcomes, despite the small sample size.

In our series, baseline itch severity differed significantly between females and males, as assessed by p-VAS and ISS (Amatya et al, 2010; Damiani et al, 2019; Junsuwan et al, 2023; Sampogna et al, 2004). Consistent with some reviews, females experienced greater QOL impairment (Janowski et al, 2014; Junsuwan et al, 2023; Morariu et al, 2024; Mrowietz et al, 2011; Reich et al, 2010; Yosipovitch et al, 2000), whereas differences in PASI and DLQI were not significant. Although not fully understood, several factors may explain sex differences in pruritus. Biological factors, including hormonal state, innervation, and skin characteristics, may enhance itch perception. Psychosomatic factors (eg, anxiety and skin-picking behavior), which are more common in females, may amplify symptoms. Social factors, such as a greater concern for physical appearance, may further heighten itch awareness in females compared with males (Czarnecka-Operacz et al, 2015; García-Fernández et al, 2019; Kursewicz et al, 2020; Murer et al, 2021; Ständer et al, 2013).

Globally, the association between clinical scores remains controversial. While some studies have underlined a correlation between PASI and pruritus intensity on p-VAS and ISS (Damiani et al, 2019; Kim et al, 2014; Szepietowski et al, 2004), others, including our own, found no such association (Czarnecka-Operacz et al, 2015; Yosipovitch et al, 2000).

Consistent with the previous literature, the scalp, trunk, and limbs were the most frequently affected sites, variably associated with clinical scores (Damiani et al, 2019; Junsuwan et al, 2023; Morariu et al, 2024; Reich et al, 2007). Specifically, scalp involvement was associated with higher PASI scores, likely to reflect its high prevalence and substantial contribution to the overall disease burden. This relationship may be driven, on the one hand, by the scalp’s dense network of sensory nerve fibers, which may enhance neuroimmune interactions and thereby promote inflammation and pruritus, and, on the other hand, by repeated mechanical stimulation, including scratching and friction, which may further aggravate disease activity. In contrast, the upper limbs were associated with greater itch severity and poorer QOL, possibly owing to increased exposure to friction, external stimuli, and easier access for scratching, which may perpetuate the itch–scratch cycle. Nonetheless, the visibility and functional importance of the upper limbs may also explain a stronger impairment of QOL. The absence of an association with trunk involvement should be interpreted cautiously, given the relatively small number of patients with trunk lesions.

Although IL-17A serum levels were not associated with any clinical score in our cohort, a significant reduction was observed after treatment. The relationship between serum IL-17A levels and PASI remains conflicting: some reports show a correlation (Arican et al, 2005; Caproni et al, 2009; Ganeva et al, 2024; Takahashi et al, 2010), whereas others do not (Kyriakou et al, 2014; Xu et al, 2022). With few exceptions (Czarnecka-Operacz et al, 2015; Yilmaz et al, 2012), most studies report significantly elevated IL-17A serum levels in patients with psoriasis compared with healthy controls (Chhabra et al, 2016; Coimbra et al, 2010; Ganeva et al, 2024). Only a limited number of studies have investigated the cutaneous distribution of IL-17A in psoriasis. According to our results, which explicitly detected the IL-17A isoform, the cytokine was predominantly found in the dermal papillae and reticular dermis, with weaker staining in the epidermis. Notably, Johnston et al, (2013) demonstrated that IL-17C is natively synthesized by keratinocytes, whereas IL-17A is strictly restricted to infiltrating immune cells located mainly in the dermis. Therefore, the scattered focal spots observed within the epidermis in our series likely emphasize an extracellular binding of IL-17A to the epidermal IL-17RA/IL-17RC receptor complex rather than endogenous production. Consistent with our findings, accumulating data indicate that neutrophils and mast cells migrate into the epidermis during plaque formation, establishing a prominent neuro-immune and neutrophil–keratinocyte crosstalk that drives early lesion development (Reich et al, 2015). Although perilesional samples were not available in our study, published data—in which IL-17A has commonly been assessed at the mRNA level rather than by immunohistochemistry—demonstrate an increased expression in lesional skin compared with perilesional or healthy skin (Johansen et al, 2009; Johnston et al, 2013; Kolbinger et al, 2017; Soderstrom et al, 2017; Tollenaere et al, 2021; Vandeghinste et al, 2018; Wilson et al, 2007; Yilmaz et al, 2012). Notably, recent evidence indicates that clinically uninvolved perilesional skin already expresses IL-17A–related “signature” genes, suggesting early activation of the IL-17A axis during lesion development. In this setting, our observation of IL-17A staining in established plaques aligns with the local tissue manifestation of this activated axis.

Moreover, although our results focused on neuro-immune mediators such as IL-31 and SP, IL-17A is known to drive psoriatic itch through indirect, epithelium-driven pruritogenic downstream pathways. It induces the expression of epithelial antimicrobial peptides, such as human β-defensin 2, which directly promotes itching via Toll-like receptor 4 and Mas-related G protein-coupled receptors signaling on cutaneous sensory neurons (Feng et al, 2017; Zhang and McNeil, 2019). While these reports delineate an indirect, epithelium-mediated pathway, our findings offer a complementary perspective by highlighting a more direct neuro-immune axis involving the interplay of IL-17A with classical pruriceptive mediators.

Notably, IL-17A levels decreased more markedly in tissue than in serum from baseline to T1. As circulating IL-17A is often difficult to quantify reliably using standard ELISA, serum changes should be interpreted cautiously, particularly in the absence of high-sensitivity assays. Together with the topical application of the foam in the absence of systemic treatment, tissue cytokine changes are likely more informative than serum variations.

In this regard, in the literature, the efficacy of calcipotriol-betamethasone foam was mainly focused on variations in tissue IL-17A levels, whereas serum levels were analyzed only in cases of systemic therapies (Germán et al, 2019; Heim et al, 2022; Kubin et al, 2017; Lovato et al, 2016; Takeoka et al, 2020).

Although no correlation was found between IL-31 serum levels and clinical scores, a significant reduction was detected at T1. Consistent with our findings, most studies report no association between IL-31 levels and either psoriasis or itch severity (Chaowattanapanit et al, 2020; Purzycka-Bohdan et al, 2021; Wongjirattikarn et al, 2024); however, Nguyen and Tran, (2025) reported a unique association between IL-31 levels and itch severity (Nguyen and Tran, 2025). These discrepancies in the literature underscore the complexity of IL-31’s role in psoriatic itch. While local administration of IL-31 is a well-established pruritogenic stimulus, its baseline cutaneous or serum levels do not consistently mirror the subjective intensity of pruritus in psoriasis. In this regard, IL-31 may act as part of a broader multi-cytokine itching network rather than a standalone driver of pruritus. To date, only 1 study has evaluated IL-31’s cutaneous distribution by immunohistochemistry in psoriasis, reporting diffuse epidermal expression and strong cytoplasmic staining in dermal inflammatory cells (Wongjirattikarn et al, 2024). In addition, in our samples, IL-31/IL-31RA expression was also detected in eccrine glands and appendages, potentially reflecting the increased sympathetic innervation and type-C fibers, together with autonomic dysfunction. Noteworthy is that eccrine sweat glands are richly innervated by neuropeptide-releasing fibers, including SP, which can directly stimulate epithelial and inflammatory cells to upregulate the IL-31/IL-31RA axis. Ultimately, this localized expression creates an anatomical and functional hub linking autonomic activity to pruritogenic signaling. Accordingly, IL-31/IL-31RA expression in eccrine structures might explain why sweating, thermal shifts, or autonomic stress exacerbate lesions, trigger flare-ups, and promote itching.

Data on IL-31 distribution remain inconsistent: some studies report increased IL-31 levels in patient serum (Bilsborough et al, 2006; Chaowattanapanit et al, 2020; Narbutt et al, 2013; Nguyen and Tran, 2025; Purzycka-Bohdan et al, 2021) and perilesional skin, (Wongjirattikarn et al, 2024), whereas others report no differences compared to healthy controls (Czarnecka-Operacz et al, 2015). These conflicting observations could stem from variations in patient cohorts, disease phenotypes, or the highly dynamic nature of cytokine tissue expression during different clinical phases. As for IL-17A, in our series, IL-31 significantly decreased posttreatment, particularly in tissue samples. This finding suggests that topical calcipotriol-betamethasone foam may indirectly reduce cytokine expression by modulating type 2 T-helper cell-inflammatory pathways. To our knowledge, this is the first study to evaluate IL-31 levels in relation to the efficacy of calcipotriol-betamethasone foam.

Under basal conditions, human keratinocytes express minimal or undetectable levels of IL-31RA. However, the psoriatic inflammatory milieu may serve as a potent trigger in upregulating the IL-31/IL-31RA axis. Notably, previous studies indicate that IL-17A, particularly in synergy with TNF-α, directly increases IL-31RA expression on keratinocytes (Beringer et al, 2018; Dillon et al, 2004). Binding of locally produced IL-31, potentially enhanced within the eccrine microenvironment, activates signal transducer and activator of transcription 3 signaling, impairing epidermal differentiation and triggering the release of pro-inflammatory chemokines and neuroimmune mediators, including SP.

Although a significant reduction in SP levels was observed after treatment, no correlation with clinical scores was detected in our cohort. Conversely, a previous study reported opposite results, with no increase in SP plasma levels compared with healthy controls and no correlation with PASI (Reich et al, 2007). Accordingly, most studies report increased SP expression in psoriasis compared with healthy controls, predominantly localized in the dermal papillae, with fewer staining in the epidermis (Al’Abadie et al, 1995; Chan et al, 1997; Jiang et al, 1998; Naukkarinen et al, 1993, 1989; Pincelli et al, 1993). However, other reports found no increase (Anand et al, 1991) or even diminished SP levels (Pincelli et al, 1993). Cutaneous sensory nerve fibers are the major physiological source of SP in the skin and display in a dense network within the superficial dermis and extend free nerve endings into the epidermis. Likewise, accumulating evidence indicates that activated immune cells, including T lymphocytes and other cells, may also express SP under inflammatory conditions. Although SP immunoreactivity in our samples was predominantly associated with the inflammatory infiltrate, the present analyses do not allow discrimination between neural and immune sources; therefore, dedicated colocalization studies will be required to clarify the origins of SP in psoriatic lesions.

In addition, several studies have found augmented expression of SP receptors in psoriatic skin (Amatya et al, 2011; Chang et al, 2007; Pincelli et al, 1993; Remröd et al, 2007; Staniek et al, 1999). Interestingly, higher SP levels have been found in mature plaques compared with early lesions, suggesting a progressive role of this neuropeptide in disease evolution (Chang et al, 2007; Naukkarinen et al, 1993). Moreover, the density of SP-positive nerves and SP receptor levels measured in lesional skin were significantly associated with p-VAS scores (Amatya et al, 2011). Similar to IL-17A and IL-31, SP reduction appeared more pronounced in tissue than in serum, although direct evidence linking SP modulation to the efficacy of calcipotriol-betamethasone foam is currently lacking.

To better characterize the immunological lesional microenvironment at baseline, we demonstrated IL-31/ IL-31RA colocalization with CD4^+^ T lymphocytes on confocal microscopy. Considering the established role of type 2 T-helper cells as one of the main promoters of the IL-31/IL-31RA pathway, our data highlight the presence of an active pruritic signaling loop in baseline psoriasis lesions. Although our results focus on pretherapeutic intervention rather than on tracking its longitudinal variation, characterizing this initial intersection provides a valuable benchmark for underscoring the complexity of the local cytokine network.

The 4-week therapy yielded a significant reduction in itching as measured by qualitative scores. The efficacy of calcipotriol-betamethasone formulations has been extensively documented, with foam demonstrating superior itch reduction compared with gel or ointments, alongside fewer medical appointments and a decreased likelihood of escalation to systemic treatment or therapy switching (Duvetorp et al, 2019; Jalili et al, 2019; Koo et al, 2016; Paul et al, 2017). Both components of calcipotriol-betamethasone may participate in reducing pruritus by modulating the cutaneous neuroimmune axis. Betamethasone suppresses T-cell activation and mast cell degranulation, reducing the release of pruritogenic cytokines such as IL-31. In parallel, calcipotriol inhibits IL-31RA expression on peripheral nerve endings and keratinocytes, together with modulating the neural microenvironment by regulating nerve growth factor and its receptor TrkA, thereby counteracting the epidermal hyperinnervation of psoriatic plaques. Together, this synergism may interrupt the itch–scratch cycle by reducing both inflammatory mediators and neuronal sensitization. Nevertheless, recent literature also acknowledges certain benefits of alternative vehicles, including cream formulation (Scribani Rossi et al, 2026). Although foam and cream achieve comparable PASI improvement, cream has shown superior itch control, tolerability, and patient satisfaction. While our data confirm the established therapeutic benefits of the foam vehicle, these findings underscore the importance of vehicle properties in optimizing symptom control, adherence, and real-world outcomes. Ultimately, our posttreatment tissue reduction of IL-17A, IL-31, and SP strongly suggests that this topical calcipotriol-betamethasone disrupts a self-amplifying loop by simultaneously suppressing both the upstream cytokine triggers and the downstream neurogenic effectors.

### Conclusion

This study provides a combined clinical and immunopathological assessment of mild-to-moderate psoriasis vulgaris, confirming the relevant burden of pruritus on patient’s QOL. Although no global correlation was observed between disease severity and pruritus scores, a notable female-to-male difference emerged, suggesting a possible sex-related modulation of itch. Selected associations emerged with BMI and lesion distribution. Treatment with calcipotriol-betamethasone foam for 4 weeks resulted in a significant decrease in cytokine levels, particularly within lesional skin, likely due to the local therapeutic effect. Although no direct correlation between cytokine expression and itch intensity was demonstrated, an indirect relationship appears plausible and warrants further investigation in larger cohorts.

## Materials and Methods

### Patients

Over a global 1-year period, 25 patients with mild-to-moderate psoriasis vulgaris attending a tertiary referral dermatology clinic were enrolled. The study was approved by the Regional Ethical Committee and conducted in accordance with the Declaration of Helsinki.

#### Inclusion and exclusion criteria

Diagnosis was confirmed by clinical and immunopathological criteria. Patients with other forms of psoriasis, concomitant skin diseases, pregnancy, or personal or familial atopic predisposition were excluded. Eligible patients provided informed consent, and enrolment was completed upon entry of the required data into the study database.

#### Patient characteristics

Demographic data—including sex, age of onset, age at diagnosis, race/ethnicity, family history of psoriasis, and BMI—were retrieved from patients' electronic health records and dedicated databases to characterize the study cohort. Concurrent therapies and comorbidities were also recorded. Patients were evaluated at baseline (T0) and after a 4**-**week treatment with calcipotriol-betamethasone foam (T1), applied once a day to affected areas without other topical or systemic treatments. Clinical evaluation included affected regions and symptom severity assessed by PASI (Fredriksson and Pettersson, 1978), DLQI (Finlay and Khan, 1994), p-VAS (Reich et al, 2012), and ISS (Majeski et al, 2007). Serum IL-17A, IL-31, and SP levels were measured by ELISA at T0 and T1 using commercially available kits. Moreover, IL-17A, IL-31/IL-31RA, and SP levels were evaluated in lesional skin by immunohistochemistry, with IL-31/IL-31RA additionally evaluated in clinically healthy perilesional tissue obtained by a 4 mm punch biopsy. Skin from healthy controls was obtained adjacent to excised benign cutaneous tumors. Immunofluorescence was additionally performed for SP, IL-31/31RA, and CD4^+^ T-cells.

### Laboratory findings

#### Immunohistochemistry

Skin sections were fixed in ice-cold acetone, rehydrated in Tris-buffered saline, and endogenous peroxidase was blocked for 10 minutes. Sections were incubated overnight with primary antibodies: anti-IL-17A (mouse mAb, 1:1000; Abcam, Cambridge, United Kingdom; catalog no. ab189377), anti-IL-31 (rabbit polyclonal, 1:50; Abcam, catalog no. ab102750), anti-IL-31RA (rabbit polyclonal, 1:100; Abcam, catalog no. ab113498), and anti-SP (mouse mAb, 1:100; Abcam, catalog no. ab14184). Sections were then incubated with secondary antibodies and EnVision+ System-HRP (Dako, Glostrup, Denmark) for 30 minutes. Chromogenic detection was performed using a substrate-chromogen solution. Sections were counterstained with Mayer's hematoxylin, cleared, and mounted. Negative controls were processed in parallel using isotype-matched IgG polyclonal and IgG2a mAbs. Two blinded observers evaluated the slides, counting positive cells in 3 consecutive 200 × fields. IL-17A, IL-31, and IL-31RA expression was determined in serial sections.

#### Immunofluorescence

Six-micrometer fresh-frozen sections were fixed in ice-cold acetone and rehydrated in PBS for 10 minutes. Sections were incubated overnight with primary antibodies: anti-IL-31 (rabbit polyclonal, 1:75; Abcam, catalog no. ab102750), anti-IL-31RA (mAb, 1:150; Abnova, catalog no. H00133396-M01), anti-SP (guinea pig polyclonal, 1:100; Abcam, catalog no. ab106291), and CD4 Alexa Fluor 488 conjugate (1:20; Invitrogen). After 2 PBS washes, Alexa Fluor 568 goat anti-rabbit IgG (1:400; Invitrogen) and Alexa Fluor 488 goat anti-mouse IgG (1:400; Invitrogen) were applied for 1 hour. Slides were washed, mounted with SlowFade Gold Antifade reagent (Invitrogen), and imaged using a Nikon C2 confocal microscope. Two independent operators evaluated the staining qualitatively and by counting positive dermal cells in 3 consecutive 200 × fields.

#### ELISA

Venous blood was collected into sterile, non-heparinized tubes, allowed to clot for 30 minutes at room temperature, and centrifuged at 1200 rpm for 10 minutes. Serum aliquots were stored at −80 °C and thawed only once prior to batch analysis. IL-31, IL-17A, and SP levels were measured using commercially available ELISA kits: human IL-31 (catalog no. BMS2041-2; Thermo Fisher Scientific, Waltham, Massachusetts, USA), human IL-17A (catalog no. D1700; R&D Systems, Minneapolis, Minnesota, USA), and human SP (catalog no. KGE007; R&D Systems). Detection limits were ∼1.2 pg/ml for IL-31, ∼15 pg/ml for IL-17A, and ∼43.8 pg/ml for SP.

### Statistical analysis

Data were analyzed using Stata version 19 (StataCorp, College Station, Texas, USA). The normality of continuous variables was assessed with the Shapiro–Wilk test. Baseline and follow-up measurements were compared using paired *t-*tests for normally distributed variables and Wilcoxon signed-rank tests for non-normally distributed variables. Associations between PASI and other clinical scores, as well as between clinical scores and cytokines (IL-17A, IL-31, and SP in serum and in lesional tissue, IL-31RA in lesional tissue only), were evaluated using Spearman or linear regression models, as appropriate. Given the limited sample size, linear regression analyses used robust standard errors. Comparisons among BMI categories (normal weight, overweight, and obese) were performed using 1-way ANOVA for normally distributed variables and the Kruskal–Wallis test for non-normally distributed variables. A 2-tailed *P*-value < .05 was considered statistically significant.

## Ethics Statement

Reviewed and approved on 07/05/2019 by Comitato Etico Regionale per la Sperimentazione Clinica della Regione Toscana, Sezione AREA VASTA CENTRO, Protocol number PSO_2018, Numero registro pareri del Comitato Etico 14626_bio. All patients provided written informed consent prior to participation.

## Data availability statement

The data underlying this article cannot be shared publicly due to reasons of sensitivity. The data will be shared on reasonable request to the corresponding author.

## ORCIDs

Irene Bonanni: https://orcid.org/0009-0007-8253-4127

Lavinia Quintarelli: https://orcid.org/0000-0002-9228-8042

Marta Donati: https://orcid.org/0000-0002-9996-2896

Alessandro Magnatta: https://orcid.org/0009-0008-1701-8772

Luca Sanna: https://orcid.org/0009-0004-0630-3501

Virginia Corti: https://orcid.org/0009-0009-3637-9845

Simone Landini: https://orcid.org/0009-0007-5416-4392

Elena Biancamaria Mariotti: https://orcid.org/0000-0003-0388-1037

Valentina Ruffo di Calabria: https://orcid.org/0000-0003-0307-6636

Alberto Corrà: https://orcid.org/0000-0003-0903-4998

Alessio Coi: https://orcid.org/0000-0002-9816-3144

Chiara Della Bella: https://orcid.org/0000-0002-2565-6220

Alice Verdelli: https://orcid.org/0000-0003-1982-888X

Marzia Caproni: https://orcid.org/0000-0002-7471-3471

## Conflict of Interest

The authors state no conflicts of interest
